# Experiences and challenges of enrolled nurses undertaking a Bachelor of Nursing Science programme in Namibia

**DOI:** 10.4102/curationis.v47i1.2549

**Published:** 2024-05-06

**Authors:** Daniel O. Ashipala, Paulus K. Kapula, Alice Lifalaza

**Affiliations:** 1 Department of General Nursing Sciences, Faculty of Health Sciences and Veterinary Medicine, University of Namibia, Rundu, Namibia

**Keywords:** advancing, Bachelor of Nursing Science, challenges, enhancing, enrolled nurse, experiences, programme

## Abstract

**Background:**

Globally, enrolled nurses (ENs) are embarking on an educational journey to become registered nurses (RNs) in order to enhance their knowledge and career opportunities. However, their aspiration is not without challenges. In Namibia, the experiences of these nurses have not been extensively researched.

**Objectives:**

This study aims to explore and describe the experiences and challenges of ENs undertaking a Bachelor of Nursing Science at the University of Namibia.

**Method:**

A qualitative, exploratory, descriptive and contextual research strategy was followed as the basis of conducting the study. A sample of 15 nursing students was purposively selected from the target population of 73 nursing students. This sample size was determined by the saturation of data as reflected in repeating themes. The collected data were analysed thematically using an inductive approach.

**Results:**

Three main themes subsequently emerged from the study: ENs’ positive experiences advancing in the Bachelor of Nursing Science (BNSc) programme; nurses’ negative experiences advancing in the BNSc programme; and recommendations to ensure effective advancement in the BNSc programme

**Conclusion:**

The findings of this study revealed that ENs positively experience becoming a RN when it comes to self-development; however, they have negative experiences such as not receiving exemptions for prior learning and having to learn a new curriculum.

**Contribution:**

These findings may be used by the Faculty of Health Sciences, School of Nursing and Public Health management in order to develop targeted interventions and ongoing strategies during their curriculum review cycles to ensure positive student experiences and success within the programme.

## Introduction

Student nurses who previously qualified as enrolled nurses (ENs) account for a significant number of the students undertaking a Bachelor of Nursing Science (BNSc) degree worldwide (Kilstoff & Rochester 2012). Without higher qualifications, the career opportunities and remuneration for ENs are likely to be poor compared to those for registered nurses (RNs). Undertaking a further study through enrolment in a BNSc programme is therefore an attractive option for ENs (Tower et al. 2015). Asiimwe, Muwema and Drake (2019) reported that studies in North America have found that BNSc graduates are contributing to improved access to health services and better health outcomes for healthcare users. Notably, acute care hospitals with higher proportions of nurses educated at the baccalaureate level or higher experience lower overall mortality rates by 5% in surgical patients who experienced serious complications. The motivation for ENs to upgrade their qualifications to become an RN appears to stem from three sources: an intrinsic drive for self-development; a reaction against the restricted nature of the existing EN role; and a decline in employment opportunities (Fields 2017).

In countries such as New Zealand and the United Kingdom, ENs looking to improve their qualifications do so because of a general lack of employment opportunities, which has been exacerbated by the present phasing out of the role. This has led to the creation of a single division of nursing, that is, registered nursing (Francis & Humphreys 2011). In Australia, even though the role remains well-supported by both the private sector and the government, the employment of ENs has declined by over 20% since the late 1980s, largely because of the restructuring of aged care services (Wall 2016).

A study conducted in South Africa by Bvumbwe and Mtshali (2018) indicated that because of the current nursing education system in South Africa, ENs who are upgrading students give up their jobs to study hence their financial difficulties. In addition, a shortage of RNs may also limit the opportunity for teaching and guiding ENs in their transition, making it more difficult for them to achieve competencies in their tasks. This compromises the learning needs of ENs. According to Woo and Newman (2020), several factors influence the transition to RN, in particular the impact of the workforce, workplace reality, knowledge, and skills expected of a new RN, the available support system, adjusting to higher levels of responsibility, and accountability.

Studies have indicated that successfully upgrading is not without its challenges (Asiimwe et al. 2019); however, unlike the majority of undergraduate nursing students, ENs enter BNSc programmes as healthcare professionals, and are therefore not given any recognition of their previous learning and experience. This positions ENs as a unique sub-cohort of students based on the wealth of prior professional knowledge and skills that they bring to the≈teaching environment. It is therefore important that tertiary institutions recognise and understand the challenges that these students experience (Wall, Fetherston & Browne 2020). Some of the major challenges related to upgrading ENs could be because of a lack of understanding of what the RN role entails, as well as the intensive training needed. When upgrading, ENs may find it challenging to become a student again; some report a sense of separateness and difference between their identity and that of other nursing students (Van Reyk et al. 2017). Enrolled nurses who are upgrading undergo a 4-year training course, which seems long given that there is repetition of previous content from their EN course. Moreover, there is no recognition for prior learning content as part of their past professional experience of the content they studied previously, and they are not given the opportunity to obtain prior learning exemptions.

According to Rapley et al. (2015), ENs who are in transition experience pedagogical and academic learning difficulties, whereby ineffective teaching methods, overcrowded classrooms, and inadequate infrastructure can lead to poor academic performance. Enrolled nurses who are upgrading are also likely to experience financial problems, which can lead to absenteeism. In addition, ENs have reported that they struggle to balance all the priorities in their lives with the demands of their studies. They experience increased stress levels because of their monetary struggles (Wall 2016). Despite the fact that this phenomenon is widely studied globally, it has not been extensively researched in the Namibian context.

Furthermore, according to a study by Wall et al. (2020), additional data have emerged from Australian studies about the EN experience within BNSc programmes (Craft et al. 2017; Fields 2017; Kenny & Duckett 2015), which have identified academic writing, learned critical thinking, and balanced competing priorities as challenges for ENs.

In Namibia, the University of Namibia recognises the need for advancing ENs to become BNSc degree holders through one of two ways: mature age entry or Recognition of Prior Learning (RPL). Once admitted, however, they are enrolled in a 4-year BNSc degree (Clinical) (Honours) programme with no exemptions. This positions ENs as a unique sub-cohort that faces challenges that other students do not experience (Wall et al. 2020). Some of these challenges include the pressure of new expectations, a lack of understanding of what the RN role entails, as well as the intensive training needed to be an RN and other family life responsibilities. Studies have also highlighted that for ENs to become RNs, they need to adapt to a new student role (Tower et al. 2015), which can be challenging.

### Aim

The aim of this study was to explore and describe the experiences and challenges facing ENs undertaking a Bachelor of Nursing Science at the University in Namibia.

## Methods

### Research design

A qualitative, explorative, descriptive, and contextual approach was used to explore and describe the lived experiences of ENs undertaking a BNSc programme at the University of Namibia, Rundu campus based on their own understandings. Qualitative research is concerned with everyday human experiences within a natural environment (Maree 2016). The descriptive design was employed for the study as it allowed the researchers to describe the phenomenon as reported by the participants (Polit & Beck 2017).

### Setting

The University of Namibia is a premier institution of higher learning in the country, with 12 satellite campuses nationwide. Of these, only four (main campus, Rundu, Oshakati, and Southern) offer a Bachelor of Nursing Science (Clinical) (Honours) degree. This study was conducted at the University of Namibia, Rundu campus, which is located in the east of Namibia.

### Population and sample

Participants in this study were second, third, and fourth-year undergraduate nursing students who were enrolled in the Bachelor of Nursing Science (Clinical) (Honours) programme at the University of Namibia, Rundu campus. In the 2020 academic year, 450 students including second, third, and fourth-year nursing students were registered in this programme at the School of Nursing and Public Health, Rundu campus of the University of Namibia. This constituted the population for this study. Enrolled nurses who are registered in this course have to resign from their jobs and they are responsible for funding their studies. The course delivery has a ratio of 50:50 theory and practice, where nursing students spend 2 weeks in class for theory and 2 weeks at clinical practice facilities (Shatimwene, Ashipala & Kamenye 2020). Non-probability purposeful sampling was employed based on the judgement of the researchers regarding those participants who would be especially knowledgeable about the research topic, as suggested by Brink, Van der Walt and Van Rensburg (2018) and Polit and Beck (2017). The selection was based on the following inclusion criteria: (1) ENs with at least 1 year as an undergraduate nursing student and enrolled in the Bachelor of Nursing Science; (2) willing to participate and sign an informed consent form; and (3) available at the time of data collection. Pilot interviews were conducted prior to the main study with three participants who previously worked as ENs, with the aim of adjusting the data-collection tool prior to the main study, if necessary. Participants who took part in the pilot study were not included in the main study. No changes were made to the interview guide. The number of students who were interviewed was determined by data saturation, as reflected in repeated themes. Data saturation was reached after 15 interviews.

### Data collection

Data for this study were collected during September 2020 using semi-structured, in-depth face-to-face interviews as a first step. The use of semi-structured interviews allowed the researchers to gather information from the nurses on their lived experiences during their education journeys within their own contexts (Creswell 2014). The researcher approached nursing students through a class WhatsApp group, where he shared and explained the purpose and nature of the study. The intention underlying the invitation letter was to find those nursing students who worked as ENs before undertaking the BNSc degree programme. Nursing students who needed clarification were requested to contact the researchers directly. Interviews were conducted at one of the lecture halls at the campus; however, participants had an option to propose a venue convenient to them. All participants who took part in the study signed the informed consent prior to the interview. A total of 15 interviews were conducted, all by one researcher, which lasted approximately 45 min each in accordance with the interview guide. The number of interviews held was determined by data saturation. The participants were probed to seek clarity on any responses that were not sufficient or detailed enough in both the initial and follow-up interviews. Additionally, field notes were taken during interviews to capture non-verbal body language clues and contextual information. All the participants were audio-recorded using a digital voice recorder with participant permission. The participants were assured of anonymity and confidentiality, with the use of pseudonyms on the research tools instead of names. No new information emerged at the end of the interview with participant number 15, and at this point, data saturation was reached. The following questions were posed:

1. Can you tell me about your experience of undertaking a Bachelor of Nursing Science as an EN at the University of Namibia?
2. In your own opinion, what measures do you think should be employed to address the challenges experienced by ENs who are undertaking a BNSc at the University of Namibia?

### Data analysis

In this study, thematic analysis was used to analyse the data by the researcher. This is deemed to be the most reliable method used in qualitative research as it is fairly systematic and allows the researcher to organise the information into themes and sub-themes (Polit & Beck 2017). Firstly, the researcher familiarised himself with all the research data by reading and re-reading all the interview transcripts before making data notes. Secondly, the researcher developed initial data codes, which informed themes and categories from the data. Moreover, the researcher identified predominant themes and categories from a list of various codes which needed to be reduced to form significant themes and categories pertinent to the data. Furthermore, he reviewed and refined all themes and categories, through eliminating themes that, after some consideration, were removed because of a lack of evidence from data. Main themes and sub-themes were finalised and named, with the researcher getting an overall understanding of what each term contained before writing up the research findings. The independent co-coder and the researcher subsequently underlined the units of meaning that related to the identified categories. These units of meaning were then placed into the major categories and sub-categories that were identified. The independent coder was requested to also analyse the data according to the thematic analysis method, independently of the researcher. The two analyses were then compared to ensure trustworthiness. The independent coder was selected because of a Doctorate in Nursing education, where a qualitative interview method was utilised. Phrases that pertained directly to the phenomenon and captured the essence of the experience were extracted as a unit of general meaning. The categories or units of meaning were clustered and those redundant were discarded. Subsequently, three themes emerged. Table 2 shows themes and sub-themes.

### Trustworthiness

The trustworthiness of the study was ensured using the four criteria outlined by De Vos et al. (2017), that is credibility, dependability, confirmability, and transferability. Credibility was achieved through probing to ensure sufficient data capturing from participants, prolonged engagement with, and persistent observation of the participants in the field. Dependability was achieved as the data were recorded and the transcripts have been kept and are available upon enquiry. Confirmability was achieved through a literature review to confirm some of the findings, and voice recordings and field notes were kept as part of the audit trail. The researcher practised an attitude of reflexivity by writing down his own experiences to ensure that the data reflected the voices of the participants and not the researchers’ own biases or perceptions. Transferability, to avoid influencing the participants’ responses, was achieved by means of a complete, detailed thick description of the research design and methodology used, as well as through the purposive selection of participants.

### Ethical considerations

The study was approved by the University of Namibia Health and Research Ethics Committee (ethical clearance number: SoNREC 13/2021), as well as the Ministry of Health and Social Services Research Committee (Ref: PKK 2021) before the study was conducted. Informed consent was sought verbally from the participating respondents and through their signing of a consent form after the benefits and risks were explained to the participants. Participation in the study was purely voluntary, and the interviewees were free to withdraw at any time, although this was not encouraged. The participants were assured of anonymity, as well as confidentiality, with the use of pseudonyms on the research tools instead of names. The data were stored safely and will be disposed of according to the university’s policies.

## Results

The objective of this study was to explore and describe the experiences and challenges faced by ENs undertaking a Bachelor of Nursing Science at the University in Namibia and to make recommendations based on the study findings to ensure the effective transition from EN to RN.

### Socio-demographic description of the study participants

A total of 15 participants were interviewed. The participants were all full-time undergraduate nursing students under the age of 50 years. Of the 15 respondents, eight were male while seven were female, with an age range of between 20 years and 40 years. The characteristics of the study participants are given in Table 1.

**TABLE 1:** Characteristics of the study participants.

Variable	Participants (*n*)
**Age (years)**	
20–30	9
31–40	2
41–50	4
**Gender**	
Male	8
Female	7
**Marital status**	
Single	11
Married	4
**Level of study**	
Level 2	7
Level 3	4
Level 4	4
**Experience as an enrolled nurse (years)**	
1–3	3
4–5	4
5 and more	8

### Presentation of the findings

The following three themes that emerged from the data analysis are indicated in Table 2: ENs’ positive experiences advancing in the BNSc programme; ENs’ negative experiences advancing in the BNSc programme; and recommendations to ensure effective advancement in the BNSc programme. Table 2 also summarises the study results that are presented in the form of themes and sub-themes.

**TABLE 2:** Themes and sub-themes that emerged from data analysis.

Themes	Sub-themes
1. ENs’ positive experiences advancing in the BNSc programme	1.1 Self-development 1.2 Learning a new curriculum
2. The ENs’ negative experiences advancing in the BNSc programme	2.1 No exemption for the recognition of prior learning 2.2 EN class performance discomfort versus pre-service students 2.3 Inadequate clinical supervision 2.4 Competing priorities 2.5 Financial difficulties
3. Recommendations to ensure effective advancement in the BNSc programme	3.1 Exemption for overlapping content 3.2 Supportive guidance

BNSc, Bachelor of Nursing Science; EN, enrolled nurse.

### Theme 1: Enrolled nurses’ positive experiences advancing in the Bachelor of Nursing Science programme

This theme reflects the participants’ motives for upgrading from an EN to an RN. Sub-themes under this theme include self-development and learning a new curriculum.

#### Sub-theme 1.1: Self-development

Most participants stated that upgrading from an EN to a RN at the university exposed them to a variety of information, including management skills and procedures such as blood transfusions, leading to a greater scope of practice:

‘We have learned how to delegate and again we have learned also on how to write off duty whereby when we were ENs we were not even allowed to write the off duties.’ (P1, male, 30 years old)

‘We were not allowed to do certain things, for example like blood transfusions and managing with emergency cases.’ (P6, female, 27 years old)

#### Sub-theme 1.2: Learning a new curriculum

Learning a new curriculum with advanced knowledge and skills was reported by participants as an exciting opportunity to acquire knowledge and skills, to enable them to develop and apply deeper learning through critical thinking. Enrolled nurses reported their experience related to curriculum transformation of the BNSc programme that is perceived to be at an advanced level versus their previous EN curriculum:

‘Learning the new curriculum has empowered me to develop analytic and problem-solving skills unlike the training we got when we were ENs.’ (P14, female, 28 years old)

Additionally, the BNSc programme curriculum was reported to have exposed EN to RN managerial responsibilities that was not covered in the EN curriculum:

‘With the new curriculum I have learned how to delegate and again we have learned also on how to write off duty whereby when we were EN we were not even allowed to write the off duties.’ (P1, male, 39 years old)

### Theme 2: The enrolled nurses’ negative experiences advancing in the Bachelor of Nursing Science programme

This theme reflects the students’ negative experiences regarding their academic transition. Most participants reported challenges related to balancing their roles and responsibilities as transitioning EN nurses. The sub-themes in this theme include: no exemption for the RPL, EN class performance discomfort versus pre-service students, inadequate clinical supervision, competing priorities, and financial difficulties.

#### Sub-theme 2.1: No exemption for the recognition of prior learning

Many of the participants raised complaints that much of the content, especially in the first year, was a repetition of things they already knew or that they did at the EN training level:

‘It’s not really necessary to start with the things which we know already … So, it’s to come and do the same things that you were doing for years, to come and do. That thing is wasting our time.’ (P15, male, 42 years old)

‘Being taught something that you know, something that you have been doing for years, for example being taught temperature, pulse, BP and respiration.’ (P11, female, 30 years old)

‘Things you know already and then you do it again, it’s like you lose interest, so … perhaps we should be covering and be taught the things that we did not cover in enrolled nursing, as we are not starting from the fresh; we are already … we are adding to the wealth of knowledge we already have.’ (P6, female, 27 years old)

‘I think it’s better if they exempt us [*from*] some subjects.’ (P8, male, 42 years old)

#### Sub-theme 2.2: Enrolled nurses class performance discomfort versus pre-service students

Some participants reported bad experiences when interacting with the pre-service students during classroom presentations, which reduced their participation in class. In addition, the participants described discomfort when their young classmates were better at expressing themselves in English, which also affected their active participation in class discussions and presentations:

‘You’re in the class with these people coming from grade 12; they are still fresh from school so it’s challenging.’ (P10, male, 30 years old)

‘If we are asked to present something in front, it might be the one from school will express fluent and fast but most of us … we use to struggle.’ (P4, male, 37 years old)

#### Sub-theme 2.3: Inadequate clinical supervision

The participants observed that there was inadequate supervision from the RNs during clinical settings, when they are perceived as being qualified staff. This observation resulted in the ENs feeling that they were being treated unfairly, with the potential of not acquiring the necessary competence versus the pre-service nursing students during the BNSc programme as they get more supervision.

The participants highlighted the fact that former ENs are not sufficiently supervised and, based on their previous experience, they are expected to complete tasks without support from the RNs:

‘Most of the times we are just left alone like to do things independently because they considered us as their fellow staff.’ (P12, female, 34 years old)

‘I think supervision should be equal between the first-year students who are straight from school and enrolled nurses, regardless of how many years the person has been working.’ (P14, female, 28 years old)

‘While they use to leave me alone anything can happen and if something happens … then, they will see me as a student; there is no one will stand for me that I am a qualified staff.’ (P7, female, 29 years old)

#### Sub-theme 2.4: Competing priorities

Some participants reported that it was not easy to balance school activities and family matters, resulting in divided attention that has an effect on their academic performance:

‘There are times you been told that you do have a test into two days but reaching home you will get that there are a lot of problems that you need to solve.’ (P9, female, 41 years old)

‘We have school kids that need to be dressed, that need to eat at home, families that need to be taken care of.’ (P2, male, 29 years old)

#### Sub-theme 2.5: Financial difficulties

The participants faced financial difficulties, as while they were financially independent prior to starting their transition to become an RN, the process exposed them to financial vulnerability as they had to resign from their EN positions to become students. The majority of participants stated that they had to depend on financial support from others:

‘For example, let me say if I need something to buy books. And now I have to call others to come in for help.’ (P1, male, 39 years old)

‘You are used [*to*] getting a salary each and every month. And then you are a student now … you are not getting anything.’ (P12, female, 34 years old)

### Theme 3: Recommendations to ensure effective advancement in the Bachelor of Nursing Science programme

This theme is a description of what the participants mentioned when they were asked to make suggestions regarding what should be done to address the challenges ENs face as they advance to become RNs.

#### Sub-theme 3.1: Exemption for overlapping content

Most participants expressed concerns with the challenging course duration; EN students were already exposed to more content of what was being taught in BNSc programme. They compared the first-year modules to what was already covered in their EN nursing course:

‘When we start in first year we actually start with Temperature, Pulse, Respiration [*TPR*], whereby it’s not really necessary to start with the things which we know already. It’s better if we could start … especially in second year so that the content can even be less.’ (P1, male, 30 years old)

The participants suggested that some modules of first-year content should be exempted through RPL, for example GNS1 and Midwifery should be exempted as the content covered in these two modules was already mastered by the ENs:

‘To exempt even at least one year because most of the things especially in the first year is just like a repetition, things that we know already.’ (P9, female, 41 years old)

‘I think it’s better if they exempt us [*from*] some subjects.’ (P11, female, 30 years old)

‘They can shorten the time span that we are coming here, three years then we can go.’ (P6, female, 27 years old)

#### Sub-theme 3.2: Supportive guidance

The participants in this study expressed their frustration with the lack of adequate supervision, and recommended equal supervision for every BNSc student to ensure equitable competence:

‘Actually, we don’t know everything, so we need supervision also like others.’ (P10, male, 30 years old)

‘In my own opinion, I think supervision should be equal between the first-year students who are straight from school and enrolled nurses.’ (P4, male, 37 years old)

## Discussion

The individual experiences of the ENs indicated that becoming an RN had the advantage of self-development, as ENs who are upgrading learn management and administrative skills such as delegation, supervision, and ordering, which are not done at the EN level. In addition, they learnt new skills and knowledge, including nursing procedures such as blood transfusions and facilitating the integration of theoretical and clinical settings. These findings are in line with Kenny and Duckett (2015), who showed that there is career diversification as well as advancement in their profession.

The study findings revealed that ENs face numerous challenges during their transition from ENs to RNs, such as discomfort when compared to pre-service students, inadequate clinical supervision, competing priorities, and financial difficulties. These findings are similar to those of Wall et al. (2020), who revealed that ENs face academic demands and struggle to find a balance between their personal and professional responsibilities. The reasons for advancing to become an RN are primarily related to the greater career, professional, and self-development opportunities afforded to RNs, including the opportunity for advancement through postgraduate qualifications, as well as through hospitals and professional organisations (Ralph et al. 2013). Moreover, at the university, one is in a position to acquire new knowledge and skills, develop and apply critical thinking, as well as develop effective reasoning. These findings are consistent with Rapley et al. (2015), who found that ENs were not included in the planning of patient care because of their limited knowledge and skills, which could be addressed through transition programmes such as the BNSc programme. Participants also pointed out that they feel uncomfortable sitting in a class with younger students, because they do not have a great understanding of what adult learning is all about, so sometimes they laugh at them. Kapur (2015) demonstrated that adult learners are generally more disciplined, independent, and well-organised compared to young learners. Furthermore, the current study revealed that ENs felt they had inadequate supervision during clinical placements, noting that they are often left alone to work independently like qualified staff on the task delegated to them, because RNs assume that they know everything. Birks et al. (2017) argued that good supervision and support in clinical placement help students to learn more effectively, because students get the opportunity to explore in detail what they encountered in the wards.

A significant challenge faced by ENs transitioning to RNs was identified as balancing family and financial commitments with studying. Most participants expressed that they experienced financial hardship, as they had resigned from their jobs in order to study. In addition, asking others for financial support made them feel uncomfortable. These findings are in line with those of Wall et al. (2020), who revealed that these students struggle to find a balance between the range of demands and competing priorities they encounter in their lives at home, work, and university. In addition, financial difficulties were also revealed as a major issue, both in terms of the cost of education as well as other expenses such as childcare, travel, and cost of living (Kenny & Duckett 2015). The participants further expressed that they had difficulty balancing study demands and household chores or responsibilities. Academic performance was also affected, for example, if they had to study for a test while dealing with problems at home. Similar findings by Wall (2016) indicated that ENs struggle to balance all the priorities in their lives with the demands of their university studies, and were reported to experience an increase in stress levels related to finances as well as difficulty getting financial assistance or a scholarship. These findings also concur with those of Tower et al. (2015), who stated that managing study demands influences whether one upgrades successfully or not.

Moreover, this study revealed that the participants’ academic interest in the module was affected by the repetition of content. The participants reported that some modules were covered in their prior studies, thus it was a waste of time repeating them. This repetition led to a loss of interest and student concentration; therefore, most participants suggested that the institution shorten the course duration and avoid content overlap. In their study, Derbyshire et al. (2023) noted that participants also felt that their experience was not always taken into consideration because of repetition in the theory, as academics were not always aware of participants’ clinical backgrounds and experience. Enrolled nurses in Fields’ (2017) study similarly stated that despite having prior knowledge and more experience in clinical areas than other BNSc nursing students, they were still required to perform within the scope of the BNSc student in clinical practice and to complete all requirements successfully in order to become eligible for registration as an RN.

### Strengths and limitations of the study

This study provided a broader understanding of the experiences and challenges of ENs undertaking a BNSc at the University of Namibia. The use of an explorative design enabled the participants to freely narrate their lived experiences with a specific focus on the issues they encountered during the transition process. This study was conducted at the university campus located in the north east of Namibia. The findings are therefore limited as they cannot be generalised to other settings although it was not our intention to generalise findings. We faced tremendous challenges of finding literatures related to ENs advancing to BNSc. Further research may be conducted using a quantitative approach using a comparative design.

## Conclusion

The study findings revealed that ENs encounter challenges when attempting to advance to become an RN, such as a lack of prior learning recognition, a long course duration, and financial difficulties. Furthermore, the ENs felt uncomfortable attending classes with pre-service students who are younger than them. The results of this study also showed that RNs provide inadequate supervision in the wards to guide and support the EN students, as they are perceived to be independent practitioners. This may have a negative impact on the academic performance and psychological well-being of these students. Based on the study findings, it is recommended that effective and efficient supervision and support should be made available in clinical settings to help students learn more effectively and develop the needed competencies. The institution could consider reducing the course duration to 3 years by exempting some modules in order to recognise the ENs’ prior studies and their professional experience in accordance with the institutional RPL policy requirements.
